# Expert-based controllability assessment of control transitions from automated to manual driving

**DOI:** 10.1016/j.mex.2018.05.007

**Published:** 2018-05-19

**Authors:** Frederik Naujoks, Katharina Wiedemann, Nadja Schömig, Oliver Jarosch, Christian Gold

**Affiliations:** a Wuerzburg Institute for Traffic Sciences, Germany; b BMW AG, Germany

**Keywords:** TOC-Rating (Take-over controllability rating), Automated driving, Conditional automation, Take-over performance, Controllability, Expert rating

## Abstract

Up to a level of full vehicle automation, drivers will have to be available as a fallback level and take back manual control of the vehicle in case of system limits or failures. Before introducing automated vehicles to the consumer market, the controllability of these control transitions has to be demonstrated. This paper presents a novel procedure for an expert-based controllability assessment of control transitions from automated to manual driving. A standardized rating scheme is developed that allows trained raters to integrate different aspects of driving performance during control transitions (e.g., quality of lateral and longitudinal control, adequateness of signalling to other road users, etc.) into one global controllability measure based on video material of the driving situation. The method is adapted from an existing assessment procedure that has been successfully applied to assess the criticality of driving situations in manual driving conditions (e.g., assessment of substance-induced impairments, assessment of fitness-to-drive of novice drivers). This paper presents the rating procedure, including instructions of how to code relevant qualities of the drivers’ performance with accompanying video-demonstrations, and material used for rater training.

• A rating procedure for an expert-based controllability assessment of control transitions from automated to manual driving based on observation of video material was adapted from an existing method used in studies on manual driving.
• The advantage of this method consists in an integration of different dimensions of driving performance (e.g., operational and tactical driving behaviour, criticality of the situation) into one global controllability measure.
• The method allows an assessment and comparison of diverse take-over scenarios, detached from driver performance variables.
• The accompanying video-based training material allows reproducible and reliable execution of the rating procedure.

## Graphical abstract

**Figure:**
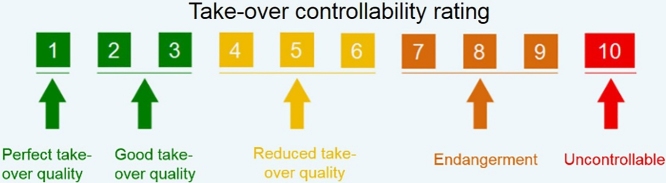

Specifications Table

**Table:** 

Subject area	• *Engineering*
More specific subject area	*Human Factors; automated driving*
Method name	*TOC-Rating (Take-over controllability rating)*
Name and reference of original method	*S.A.F.E.-rating: Kaussner, Y., Kenntner-Mabiala, R., Hoffmann, S., Klatt, J., Tracik, F., & Krüger, H.-P. (2010). Effects of oxcarbazepine and carbamazepine on driving ability: a double-blind, randomized crossover trial with healthy volunteers. Psychopharmacology, 210(1), 53-63.*
Resource availability	www.toc[HYPHEN]rating.de/en/

## Method details

### Overview of the method

The method represents a standardized rating scheme for the controllability assessment of take-over situations (“TOC-Rating”) for conditionally automated driving (SAE L3; [1]), which allows trained raters to integrate different aspects of driver’s performance during control transitions (e.g., quality of lateral and longitudinal control, adequateness of signalling to other road users, etc.) into one global controllability measure, based on video material of the driving situation and driver’s behaviour. The proposed method enables a holistic assessment of the controllability rather than an interpretation of isolated performance variables like the take-over time or lateral acceleration, which may lead to inconclusive controllability assessments. For example, Gold et al. [2] found later take-over reactions when the available time-budget was higher. However, drivers also showed a higher take-over quality, with lower accelerations. There is room for interpretation whether later but better reactions are favourable regarding controllability.

We adapted a method for the safety assessment of driving behaviour proposed by Kaussner et al. [3] to the assessment of controllability of control transitions from automated to manual driving. The method can be used in the context of empirical studies on controllability (e.g., simulator or test track studies) in accordance with the Code of Practice ([4]; ISO 26262-3, [5]). Based on a standardized rating scheme, trained raters assess the controllability of the test situations by coding participants’ performance before, during and after they have taken over manual vehicle control using video footage of the situations.

**Fig. 1:**
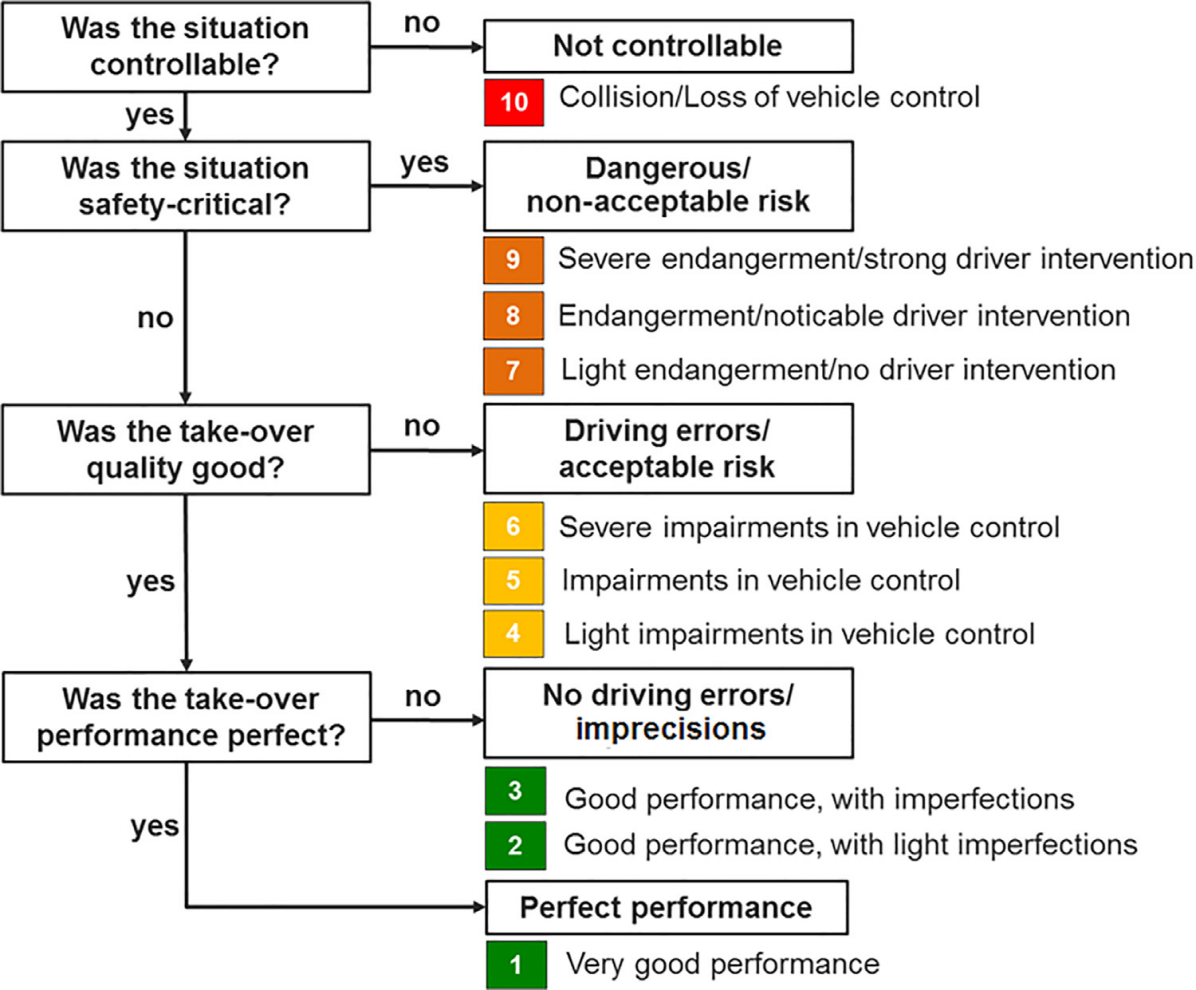
Overview of the rating process.

Imprecisions in vehicle handling, driving errors, endangerments and non-controllable events are distinguished in the coding scheme. The assessment also includes relevant aspects of the surrounding traffic (e.g., whether other traffic participants are affected) and the system’s behaviour (e.g., if the automation initiated an emergency braking manoeuvre as a result of driver inactivity). In accordance to the Code of Practice, the rating of the controllability covers the “likelihood that the driver can cope with the driving situation” [4] under consideration of the system and its limits. As a result, a comprehensive controllability rating on a scale from 1 to 10 is given, based on the frequency and severity of the coded events. The rating procedure follows a hierarchical process (see Fig. 1):

• First it has to be evaluated whether a non-controllable event has happened (e.g., collision or loss of vehicle control, which results in a rating of “10” (not controllable)). These events are specified in “*Description of the coding scheme*”.
• If this is not the case, the rater has to evaluate in a second step whether the situation has been safety-critical (i.e., the driver has endangered her/himself or other road users, involving a non-acceptable risk for the driver or other road users such as causing a near-crash situation), which results in a rating of “7-9”, depending on the level of criticality and intensity of driver intervention necessary to prevent a collision (see “*Description of the coding scheme*”).
• If the situation is not safety-critical it has to be assessed whether the take-over quality was good or not. An impaired take-over quality is defined by the occurrence of driving errors such as lane exceedances, insufficient securing or inadequate speed, whose occurrence results in a rating between 4-6 depending on the frequency and severity of the errors. The definition of driving errors is specified in “*Description of the coding scheme*”.
• In a last step, if no driving errors occurred, it has to be evaluated if the take-over performance contained any imprecisions such as imprecise lane keeping or hesitant deactivation of automation, which would lead to a rating of 2 or 3 depending on the severity and frequency. If the take-over performance was perfect (i.e., no imprecisions were observed) a rating of 1 is given.

**Fig. 2:**
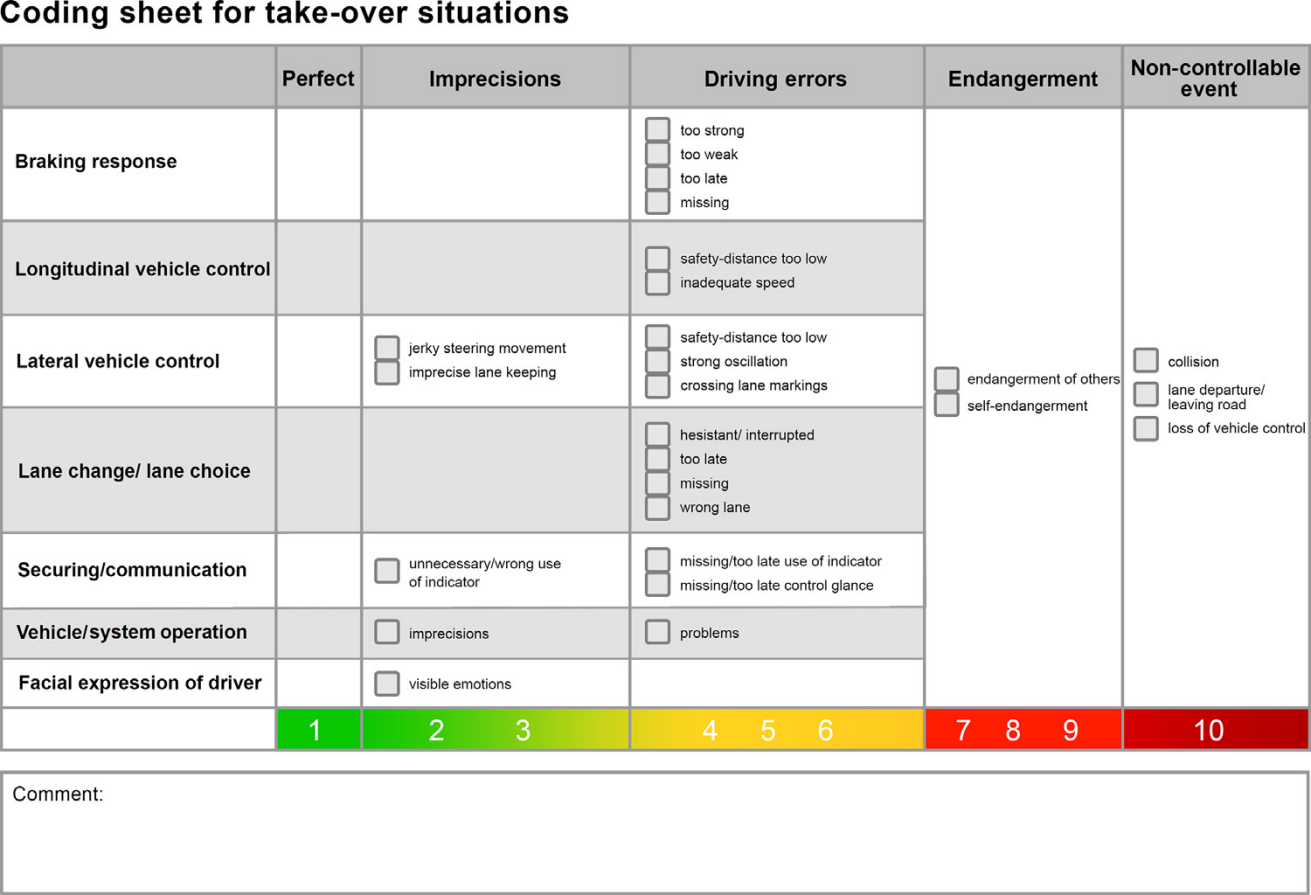
Coding sheet used for analysing video footage of the take-over situations.

Imprecisions and driving errors are related to different aspects of the driving task. In the TOC-rating, they are assessed on different categories such as braking, longitudinal control, lateral control, lane changes, communication with other road users, vehicle operation and facial expression. A coding sheet listing all coding events is used to guide the rating (see Fig. 2).

### Rating material

**Fig. 3:**
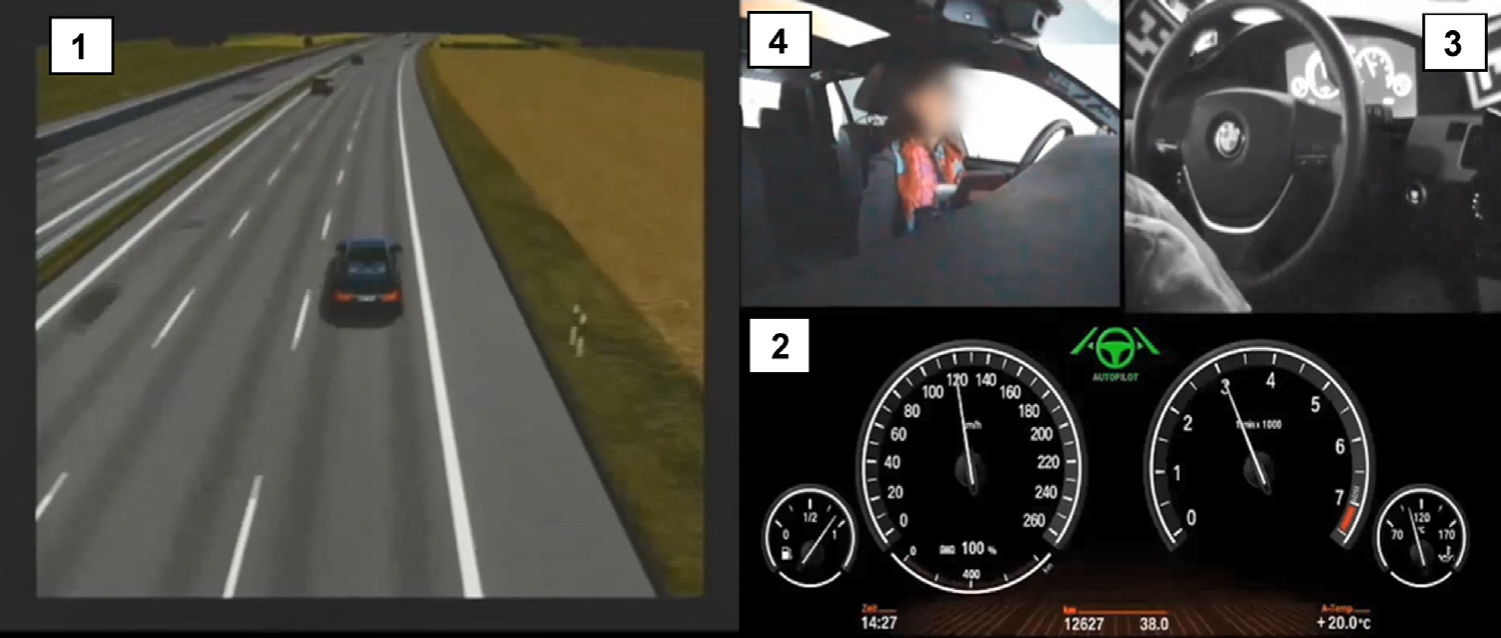
Example for video footage containing driving scene (1), HMI elements (2), control elements (3) and driver (4).

The rating material is video footage of control transitions from automated to manual driving derived from an already conducted study or explicitly collected with the aim to use the TOC-rating tool. Most often, these videos will show how the driver reacts to a request to take back manual vehicle control and how she/he executes the necessary driving manoeuvre (such as avoiding an obstacle on the road or keeping the vehicle in the lane). The video material should contain the following views (see Fig. 3):

• *View of the driving scene*: optimally both from a bird’s eye view (in order to estimate lateral and longitudinal distances and vehicle control) and from the driver’s perspective (view 1 in Fig. 3)
• *View of the HMI elements* (e.g. cluster display): necessary to define the moment a take-over request is triggered (view 2 in Fig. 3)
• *View of control elements*: necessary to estimate the driver’s intensity of steering interventions, braking interventions and the vehicle operation (e.g., one or two hands on the steering wheel) (view 3 in Fig. 3)
• *View of the driver and additional driver activities* (optional): Useful to evaluate whether and where the driver is engaged in other non-driving related activities; driver’s facial expression can be indicative of the experienced workload or surprise by the take-over situation (view 4 in Fig. 3)

Beside the visual information the videos should contain an audio track to determine the moment a take-over request is triggered, especially if the view to HMI elements is not available or temporarily obstructed. This audio track can be further used to collect comments of the driver that might give possible explanations for a specific driving behaviour and driver’s understanding of the situation.

In order to facilitate the rating process, it is recommended to cut the relevant take-over situations out of the complete video of the drive, starting at or just before the take-over request is triggered and ending at a previously specified time the take-over process has been defined as finalized by the experimenter.

The quality of the video footage will highly influence the reliability of the outcome of the rating tool as the rating is mainly based on observation of driver’s and system’s behaviour in a specific take-over scenario.

### Description of the coding scheme

The following paragraphs describe the assessment criteria and traffic events, as well as the events that are coded from the video using the hierarchical process which starts with the question if a situation has been controllable or not.

It is important to emphasize that the coding is not only depending on the behaviour of the driver, but also on the behaviour of the automated driving system and the surrounding traffic. This decision has important consequences on the rating process. For example, when the available time budget or situational constraints leave the driver no choice but to perform a strong braking manoeuvre, this is still coded as a driving error. If the deactivation of the automated driving system affords a rapid steering intervention which impairs lane keeping, this is coded as a driving error, even if it cannot be prevented by the driver in any way. Consequently, in accordance to the Code of Practice, if the constraints of the take-over situation or system inherently lead to endangerments, these are coded as such, even if the driver reacts as fast as possible. The controllability is determined by the driver, the situation and the actual system application, with all their limits and characteristics.

#### Non-controllable events

At the beginning, it is decided whether the situation was controllable or not. Non-controllable events (rating of 10) are defined in Table 1.

**Table 1:** Non-controllable events.

Coding event	TOC-Rating	Description
Collision	10	Colliding with another road user or object
Leaving the road	10	Vehicle leaves the paved part of the road with center of gravity
Loss of vehicle control	10	Driver loses control over the vehicle, leading to skidding, rotating or swerving across several lanes; this category can also be a previously defined fail-criterion indicating loss of vehicle control

#### Safety-critical events: Endangerment

In the next step, it is decided whether the driver endangered him/herself or another road user. Endangerments are defined as situations with inacceptable risk for the driver (self-endangerment) or other road users. The rating should include both the severity of the endangerment, defined as the degree to which the spatial and time safety distance to other road users or objects was undercut and the intensity of driver intervention in order to prevent a collision. This distinction is based on classifications used in naturalistic driving studies [6]. If both aspects are comparably high, the more severe criterion should be considered for the rating.

The decision whether an endangerment took place can be subjective and depending on the rater’s judgment. This deliberate subjectivity in the rating is considered an advantage rather than a limitation of the method, as it allows for adaptability to the specific scenario and situational circumstances. The following guidelines presented in Table 2 are used to guide the rating process.

**Table 2:** Endangerments (based on the definition used by Klauer et al. [6]).

Coding event	TOC-Rating	Description
Severe endangerment (“near crash”)	9	Strong undercut of safety distance **OR** Minor or moderate undercut of safety distance **AND** execution of an emergency intervention (lateral/longitudinal vehicle handling at the boundaries of driver’s capabilities)
Endangerment (“crash relevant conflict”)	8	Moderate undercut of safety distance **OR** minor undercut of safety distance **AND** driver’s intervention exceeds the standard range
Minor endangerment (“proximity conflict”)	7	Minor undercut of safety distance **AND** absent or weak driver intervention

#### Driving errors and imprecisions of vehicle handling

When the event was neither non-controllable, nor safety-critical, the raters look for driving errors or imprecisions of vehicle handling when taking back manual vehicle control. Errors and imprecisions are used to differentiate between situations that were not safety-critical, but the take-over quality was still impaired. Nevertheless, they can also be used to explain the occurrence of non-controllable or safety-critical events.

In this category the events are partly subdivided into “imprecisions” which are lapses in vehicle handling without severe consequences for the take-over performance, and “driving errors” with considerable impairments in take-over performance. Imprecisions of vehicle handling and driving errors are classified into the following categories:

• Braking response
• Longitudinal vehicle control
• Lateral vehicle control
• Lane change/lane choice
• Securing/communication
• Vehicle/system operation
• Driver’s facial expression

The *braking response* is defined as deficient if the use of the brake pedal in reaction to the situation is not adequate (too weak, too strong, too late or missing). Table 3 defines driving errors associated with braking responses.

**Table 3:** Driving errors associated with braking response.

Coding event	TOC-Rating	Description
Too strong	≥4	The level of deceleration is too high with the consequence of an unnecessary high loss of speed and/or a braking reaction, possibly down to a complete standstill.
Too weak	≥4	The level of deceleration is too low with the consequence of a rapid approach towards an obstacle or even an endangerment. The driver is forced to execute a strong braking response or an additional evasion manoeuver in order to avoid negative consequences.
Too late	≥4	The braking response is performed too late (in relation to an obstacle) with the consequence of an undercut of the safety distance. The driver is forced to execute a strong braking response or an additional evasion manoeuver in order to avoid negative consequences.
Missing	≥4	Necessary braking response is not executed with the consequence of inadequate speed, undercut of safety distance, endangerment, collision or wrong lane error (if a lane change is executed instead of braking; see below).

The *longitudinal vehicle control* is defined as deficient if either distance keeping or speed choice is inadequate. Table 4 defines driving errors associated with the longitudinal vehicle control.

**Table 4:** Driving errors associated with longitudinal vehicle control.

Coding event	TOC-Rating	Description
Undercut of safety distance	≥4	The safety distance to a vehicle/obstacle in front or behind the ego-vehicle is undercut. The error can occur as a reason for an endangerment but also as a single event without endangerment. In the latter case, the headway is not large enough to serve as an effective safety buffer, but this is not seen as an immediate endangerment.
Inadequate speed	≥4	Depending on the situation, inadequate speed can either be too high (i.e., in consequence of late, weak or missing braking response or a violation of a speed limit) or too low (i.e., in consequence of a strong braking response or an extreme undercut of the speed limit). The error should also be coded if a following vehicle is forced to brake due to the slow speed of the ego-vehicle.

The *lateral vehicle control* is defined as deficient if lane keeping or the driver’s steering response is inadequate. In this category events can be coded as imprecisions or driving errors. These are defined in Table 5.

**Table 5:** Driving errors and imprecisions associated with lateral vehicle control.

Coding event	TOC-Rating	Description
**Imprecisions**		
Jerky steering movement	≥2	The driver executes a strong and fast steering reaction either to one or both sides of the lane with the potential consequence of impaired lane keeping. This event should also be coded if it occurred in the course of a system deactivation that affords such a strong steering wheel movement.
Imprecise lane keeping	≥2	Inaccurate, imperfect lane keeping without touching the lane markings. The drift can be towards one or both sides of the lane.

**Errors**		
Strong oscillation	≥4	Inaccurate, imperfect lane keeping with stronger deviations from the middle of the lane than imprecisions; vehicle approaches lane markings; the vehicle drifts towards one or both sides of the lane approaching or touching the lane markings with the tires without crossing them.
Crossing lane markings	≥4	Vehicle crosses lane markings with the tires when no lane change was intended (i.e., vehicle drives back to the initially followed lane).
Undercut of lateral safety distance	≥4	The safety distance towards an obstacle or other road user is undercut in lateral direction; The error can occur as a reason for an endangerment but also as a single event without endangerment.

Errors in *lane change or lane choice* are coded when the driving errors relate to the initiation or execution of a lane change or the driver is following the wrong lane. The coding events are depicted in Table 6.

**Table 6:** Driving errors associated with lane change or lane choice.

Coding event	TOC-Rating	Description
Hesitant lane change/interrupted lane change	≥4	A lane change is hesitant if the driver shows unassertive behaviour during a lane change, i.e., a lane change is executed very slowly or driver waits very long until an already announced lane change (e.g., by indicating, checking the mirrors, etc.) is finally executed. A lane change is interrupted if an already initiated lane change (e.g., by indicating, approaching the dedicated lane) is cancelled.
Late lane change	≥4	Driver initiates the lane change late in spatial relation to the reason for that event (e.g., an obstacle on the lane).
Missing lane change	≥4	Necessary and possible lane change (i.e., without endangering other road users or violating their right of way) is not executed resulting in a stop in the lane (e.g., in front of an obstacle).
Wrong lane	≥4	Vehicle drives on the wrong lane according to traffic rules; (e.g., violating the German highway code by not driving on the (unoccupied) right lane; driving on the hard shoulder; overtaking on the right). Can occur as a consequence of a missing lane change or an unnecessary lane change.

Events of the driver’s *securing and communication behaviour* can be coded as imprecisions (or lapses) or driving errors. The definition of the coding events is shown in Table 7.

**Table 7:** Driving errors and imprecisions associated with securing and communication behaviour.

Coding event	TOC-Rating	Description
**Imprecisions**		
Unnecessary use of the indicator/wrong use of the indicator	≥2	Driver uses the indicator without a reason, e.g. although the lane is not changed or uses it in the wrong direction when the lane is changed; Can occur accidentally when mistaking the indicator for the control unit of system deactivation or as a consequence of problems in indicator handling.

**Errors**		
Missing use of indicators/too late use of indicators	≥4	Driver fails to indicate in case of an intended lane change or indicates too late (i.e., only after a lane change is initiated), resulting in the announcing function of the indicator no longer being fulfilled. Dependent on the presence of surrounding traffic, the severity of the error can be adapted.
Missing control glance/too late control glance in the mirrors/to the neighbouring lane	≥4	Driver fails to execute a control glance (either in the mirror, to the road or the neighbouring lanes) before executing a lane change or executes it too late, resulting in the securing function of the glance no longer being fulfilled. Dependent on the presence of surrounding traffic, the severity of the error can be adapted.

Events of *vehicle or system operations* are also subdivided into imprecisions and errors, which are listed in Table 8. Various kinds of imprecisions and errors are summarized under these two categories.

**Table 8:** Driving errors and imprecisions associated with vehicle/system operation.

Coding event	TOC-Rating	Description
**Imprecisions: Imperfections at handling vehicle and system control**		
Single-handed take-over	≥2	Driver uses only one hand when taking over manual vehicle control; the other hand can be free or occupied
Take-over with occupied hand(s)	≥2	At least one hand is occupied when driver takes over manual vehicle control (e.g., holding a portable device)
Uncertainties at solving the take-over situation	≥2	Driver is insecure how to deactivate the system, e.g., problems in finding the correct control unit but chooses the right deactivation method after intensive searching. Visible deliberations between accelerator or braking pedal before finally the correct action is taken.

**Errors: Problems at handling vehicle and system control**		
Unnecessary/unnecessary strong use of pedals	≥4	Driver shows problems in pedal usage, e.g. hits the accelerator pedal instead of the braking pedal; hits both pedals simultaneously; clear indications of false assumptions with respect to methods for system deactivation (e.g., system can be deactivated by pedal press although this is not the case). If the driver initially choses the wrong (inadequate for the respective situation) action after system deactivation, e.g., accelerates or brakes too fast; if the driver shows clear uncertainties on the correct action to choose in reaction to a specific situation (e.g., using the accelerator or brake pedal in order to solve the situation)
Problems deactivating the system	≥4	Driver is (initially) not successful at system deactivation since the respective method was not applied correctly ; examples depend on the respective system: button press too short, wrong button is pressed (e.g. indicator), one instead of two buttons is pressed; steering movement not strong enough, press of brake pedal too weak, accelerator pedal used instead of brake pedal etc.

Driver’s *facial expression* should be seen as a supplementary category, which can give valuable explanations for some unclear behaviour/patterns in take-over performance. However, they should be considered on a lower priority level and not before the other aspects have been rated, as the main basis for the rating must remain the observable take-over performance. Therefore the coding events listed in Table 9 are all categorized into the imprecision category.

**Table 9:** mprecisions associated with facial expressions.

Coding event	TOC-Rating	Description
Nervous/tense	≥2	Driver shows signs of nervousness/tension; (e.g., clenching their teeth, biting their lip; concentrated face)
Surprised/worried	≥2	Driver seems to be surprised/worried (e.g., eyes wide open, open mouth, utterances such as “oh”, “oops”)
Hectic	≥2	Driver reacts to take-over request in a hectic manner (e.g., by dropping the portable device)
Uncertain/confused	≥2	Driver shows signs of insecurity/confusion about what is happening and what they are supposed to do

### Rating process

Before starting the rating process, all raters should complete a rater training (see chapter “*Rater training*”). The next step is to provide the raters with information about the basic functionality of the system and its behaviour in a take-over situation. This for example includes the knowledge about deactivation possibilities of the system (e.g., which steering wheel button can be used for deactivation). Another important factor is to provide the raters with an “ideal solution” of the situation (e.g., that a lane change is the best solution and can be performed without interference with the neighbouring lane if the driver reacts early enough to the take-over request). Furthermore, raters must know the time window in which they should rate the take-over performance. Especially the definition of the end of the take-over process is of importance and preferably corresponds to the end of the respective video sequence. For example, in case of a take-over before a construction site, it has to be defined whether the complete manual drive through the construction site should be considered in the rating or only the immediate reaction to the take-over request in form of a lane change.

Each of the above-described events that were observed during the take-over sequence should be coded in the rating sheet in the respective category (driving errors and imprecisions either as basis for the rating or as explanations for the higher categories of endangerments or non-controllable events if one of these categories had been coded in the hierarchical rating process first). For example one event might occur as a consequence of another timely or causal preceding event, such as inadequate speed due to a too weak braking response. Both events are to be coded.

The rater is allowed and encouraged to watch the video several times and initially consider only the driving performance. In a further step, the rater can include driver’s facial expression or verbal comments for a refinement of the rating. The rater is instructed to use the rating sheet in accordance to the above described hierarchical method. If a situation was defined as dangerous or not controllable, it is recommended to also rate driving errors or imprecisions that might have led to the endangerment. The rater is further encouraged to make comments that are able to explain a specific behaviour of the driver that otherwise would remain unclear.

### From event coding to controllability rating

After imprecisions, driving errors, endangerments and non-controllable situations have been coded and categories have been checked in the video material, an integrated rating is given. The raters must keep in mind to rate the combined driver-vehicle behaviour. For example, when a take-over situation is per se very critical as the system is not able to perceive the system limit early enough, forcing the driver to endanger her/himself or other road users, this must result in a low controllability assessment (meaning high rating values in the TOC-rating) even if the driver does his/her best to solve the situation.

To arrive at a final rating, the raters should weigh the severity of the coded events, taking into account the situational context. For example, whether there is additional surrounding traffic present or not should be taken into account when assessing a situation in which the driver has changed lanes without looking into the side mirror. Note, however, that it is important to not drift into “what would have happened if there had been” ratings. Only the observable behaviour in the given situation should be rated, not potential hazards that actually have not been there.

In general, the accumulation of single events to one global controllability rating on the 10-point rating scale should lie in a certain scope of discretion defined by the raters, based on their experience. The following rules, however, are to be used as guidelines for the raters to reach their final decision:

• if at least one event of the non-controllable event category is coded: Rating = 10
• if at least one event of the endangerment category is coded: Rating ≥7
• if at least one event in the error category is coded: Rating ≥4
• if at least one event in the imprecision category is coded: Rating ≥2
• if no event is coded: Rating = 1

Within each rating category (2–3; 4–6; 7–9), the number and severity of coded events define the final rating, based on the scope of discretion of the raters. This means for example, that even if only imprecisions were coded, but of a remarkable amount, the rater is allowed and encouraged to give a rating in the range of 4–6.

### Rater training

All raters that use the TOC-Rating tool should be trained in advance by using the freely available training material available online at the following link: www.toc[HYPHEN]rating.de/enwww.toc[HYPHEN]rating.de/en. The rating material consists of a power point presentation, in which the aim of the rating and the rating principles are described (in accordance with this paper). Every rating event is clearly defined and supplemented by video examples. Though, it is recommended that the training is given by an experienced user of the method. It takes around three hours and can be performed within a group of up to ten raters.

The goal of the training is to ensure a high internal validity of the tool. Internal validity, in this case, means that all raters must have understood the criteria and rules to come to the final rating. A reliable indicator for this is a high inter-rater reliability. At the end of the training, it is recommended to evaluate this inter-rater reliability for a small set of situations (around 10 situations) from video material that is comparable to the videos that will be rated after the training. For this training set, an ideal solution should be prepared, which can be discussed with all involved raters to get a better understanding of the coding events and to increase inter-rater reliability. To the authors’ experience, this takes around two hours.

As measure for the inter-rater reliability the weighted kappa is recommended. This analysis is available in statistical tools such as SPSS (by IBM). A formula for the calculation can be found in Bortz [7]. According to Landis and Koch [8] a kappa between 0.2 and 0.4 can be defined as fair, between 0.4 and 0.6 as moderate, between 0.6 and 0.8 as substantial and between 0.8 and 1.0 as almost perfect reliability. For the TOC-rating an inter-rater-reliability >0.6 should be achieved after the rater training.

## Method validation

### Iterative optimization

The method was initially validated and optimized in a study with five raters. These were all employees of the WIVW (Wuerzburg Institute for Traffic Sciences) that had experience with the original version of the rating tool for manual driving [3]. This first-stage validation was based on an earlier version of the rating sheet, which included a higher number of coding events and a slightly different assignment of events to either the imprecision or the error category. All five raters were briefed on the rating (i.e., the rating procedure and categories were explained) and then rated a set of 10 videos of take-over situations taken from the WIVW driving simulator in order to practice the rating. Their ratings were discussed together with the trainer.

After that, they gave TOC-ratings of 45 video sequences of driver’s take-over performance during transitions from conditionally automated to manual driving. The video sequences were taken from different driving simulator studies. The take-over scenarios required either a braking manoeuver, a lane change manoeuver in order to avoid a collision with another road user or obstacle, or stabilizing the vehicle in the lane. All take-over requests were triggered visually and auditory. Half of the videos were taken from a internal BMW study conducted at the BMW driving simulator. The other half of the videos were taken from a simulator study conducted at the WIVW driving simulator by order of BMW. Drivers were engaged in a non-driving-related task (NDRT) in the moment of the take-over request in all situations.

The inter-rater reliability was calculated as weighted kappa for each pair of raters both for the rating on the 10-point scale as well as for the five category-ratings “perfect” (rating = 1), “imprecision” (rating = 2–3), “error” (rating = 4–6), “endangerment” (rating = 7–9) and “uncontrollable event” (rating = 10). The rating on the 10-point scale showed an average weighted kappa of 0.44, which can be classified as moderate [8]. The lowest kappa-values were found for the pairs where rater 1 was involved (values lower than 0.4). This rater obviously rated with less accuracy as they missed some of the relevant error events. Excluding rater 1 resulted in an average kappa of 0.50. The calculation of the inter-rater reliability for the five categories revealed comparable results with an average kappa of 0.51 (after exclusion of rater 1). Ensuring a thorough rater selection and training constitutes a key factor and determines reliability and validity of the derived results. As long as the raters fulfil the requirements, the rating provides a robust controllability assessment.

Based on a detailed analysis of the coded events and the feedback of the raters, the method was optimized in different ways. For example, some events were partly re-classified, some events were summarized into more global categories, more clear definitions were included into the rater training and rating guidelines were formulated more clearly. After this optimization, the rating was repeated with two of the five raters. They rated the 45 video sequences again with an updated version of the rating sheet. The result was a weighted kappa of 0.88 for the five category-rating which can be classified as almost perfect reliability.

### Validation study

To further validate the method and to verify the external validity of the TOC-rating tool, correlations between objective driving performance measures and the TOC-ratings were calculated. Therefore, data from an experiment conducted in a motion-based driving simulator at the BMW facilities was used. Originally, the aim of the experiment was to investigate the impact of different NDRTs on drivers’ take-over performance. The study was designed as a between-subjects experiment with the NDRT as between-subject factor. The sample consisted of 66 participants. During a conditionally automated ride, participants had to deal with different NDRTs that were aimed at influencing the drivers’ sleepiness level (*n* = 33 participants per group, see Jarosch et al. [9] for a more detailed description of the NDRT conditions).

• The first NDRT, a quiz-task, was designed to keep the driver in an adequate arousal state
• The other NDRT was designed to induce sleepiness and consisted of a monotone vigilance task.

After 50 min of conditionally automated driving, a take-over situation appeared. The scenario consisted of an accident on the lane the participants were driving in. At the moment of the take-over request, the time-to-collision with the broken down vehicle (time budget) was seven seconds. With the take-over request, the automated vehicle control was discontinued and the participants had to regain control of the car. For more information to this study see Jarosch et al. [22] (*in preparation, 2018*).

All take-over situations were extracted from the videos and handed to three raters that differed from the raters used for optimization. The raters had completed the rater training as referenced in section “Rater Training” (i.e., they were informed about the rating procedure and the rating categories). The different NDRTs were pixelated, so it was not visible which NDRT the participants had to deal with during the ride. All videos contained the birds’ eye view on the road, view on the pedals, view on the face of the driver, and the HMI.

To assess rating reliability, weighted kappa was calculated. The inter-rater reliability for the five categories revealed an average kappa of 0.67, which in reference to Landis and Koch [8] is substantial. In 6 of the 66 cases, the ratings diverged more than three points on the rating scale. In these cases, inconsistency between the raters were discussed to identify the cause of the diverging score. Afterwards, for each take-over situation, mean scores of the three raters were calculated.

To verify the external validity of the TOC-rating, correlations between the averaged TOC-scores and different driving-performance parameters were examined. Typical driving performance parameters used to evaluate the human drivers’ performance in a take-over situation are time-based measures (reaction times of the human driver; e.g. hands-on time, first breaking reaction) and quality based measures (such as maximum longitudinal/lateral acceleration). The results suggest that the TOC-ratings correlates significantly (first braking reaction) and highly significantly (e.g., steering-wheel velocity, longitudinal acceleration and lateral acceleration) with driving different performance measures (see Table 10). This indicates a high validity of the TOC-rating method.

**Table 10:** Pearson correlation between TOC-rating and driving performance measures (significant*, highly significant**).

Driving performance measure	Description	*r*	*p*
Time-based performance parameters			
Hands-on time [s]	Time between TOR and driver putting at least one hand on the steering wheel	0.224	.070
First steering reaction [s]	Time between TOR and first steering wheel movement above two degrees	−0.125	.318
First touch on brake [s]*	Time between TOR and driver putting foot on the brake pedal	0.274	.026
First braking intervention**	Time between TOR and driver depressing the brake pedal more than 10 %	0.334	.006

Quality-based performance parameters			
Steering-wheel velocity [°/s]**	Maximum steering wheel velocity	0.343	.005
Longitudinal acceleration**	Maximum longitudinal acceleration (absolute value)	0.274	.002
Lateral acceleration**	Maximum lateral acceleration (absolute value)	0.666	.001

## Acknowledgements

This work results from the joint project Ko-HAF – Cooperative Highly Automated Driving and has been funded by the Federal Ministry for Economic Affairs and Energy based on a resolution of the German Bundestag. We also thank the reviewers for their valuable time and effort.

## Additional information

### Background

Conditionally automated driving (SAE L3) is expected to be ready for introduction to the consumer market in the near future. This automation level does not afford the driver to monitor the driving situation continuously, but he/she is still required to take over manual control when requested by the automation. Concerns have been expressed that drivers might not always be able to handle such control transitions safely, because of negative side effects of automation such as fatigue, loss of situation awareness, or increased workload caused by the execution of non-driving related tasks.

To ensure a safe use of such systems, the controllability of take-over situations has thus to be ensured, before making these automated driving features available to the general public [4]. This paper presents an expert-based method that allows trained raters to assess the controllability of transitions from conditional automation (SAE L3) to manual driving (SAE L0) by adapting an established rating method that has been successfully applied to the safety assessment of manual driving [3,10,11].

### Rationale

In accordance with the Response Code of Practice (CoP; [4]), controllability of transitions to manual driving can be assessed by evaluating the safety of the traffic situation (cf. [12,13]). In previous research, different parameters that can be used to describe and assess human performance during take-over situations, such as take-over time (e.g., time until take-over is accomplished, time until first gaze on the road, etc.) and quality (e.g., minimum time-to-collision (TTC), maximum deceleration, standard deviation of lateral position, etc.) have been proposed. In theory, these can be used to differentiate safe and unsafe events (so-called safety-critical events, SCEs; [6]) by applying threshold-values. However, deciding whether a take-over situation was safety-critical on the basis of a single performance metric might be problematic for several reasons:

• First, differentiating events on the basis of threshold-values alone has been a controversial issue in the past. For example, a TTC-threshold of 1 s was originally formulated by Hayward [14] in order to distinguish between so-called ‘near-misses’ and safe driving situations. Van der Horst [15] and Hydén and Linderholm [16] proposed a comparable threshold of 1.5 s. Higher thresholds have been put forward by other researchers (e.g., [17]: 3s; [18]: 4s; [19]: 2.5s). The selection of appropriate threshold-values for other driving performance criteria such as maximum longitudinal deceleration or lateral acceleration are subject to the same constraints.
• Second, a comprehensive assessment of traffic safety might also afford the combination of different parameters such as low TTC-values *and* the occurrence of hard braking to reliably define SCEs [20]. This is especially the case since threshold values (e.g., TTC) are often reached even in non-critical situations. Assessing the safety of such situations by looking at single performance parameters may be hard to accomplish.
• Third, a truly comprehensive assessment of human performance might afford to not only take single parameters of driving performance into account, but also relate them to different levels of driving behaviour that could be impaired, such as operational and tactical aspects of driving behaviour [21].

Expert-based assessments of controllability of traffic situations are a promising solution to these challenges. Kaussner et al. [3] proposed an expert-based assessment of fitness-to-drive for the assessment of substance-induced impairments of driving behaviour. Trained raters assess different aspects of driving behaviour (e.g., operational errors such as imprecise lane keeping, tactical errors such as inadequate choice of speed, securing behaviour and adherence to traffic rules) and then aggregate the ratings afterwards to one global measure of driving performance. This proposed method adapts this procedure to the case of automated driving.
